# King Edward's Hospital Fund for London

**Published:** 1903-02-28

**Authors:** 


### HOSPITAL ADMINISTRATION.

### CONSTRUCTION AND ECONOMICS.

### KING EDWARD'S HOSPITAL FUND FOR LONDON.

The annual meeting of the General Council of King Edward's Hospital Fund for London was held at York House, St. James's, the Prince of Wales being in the chair.

Those present were:—The Rev. T. Bowman Stephenson, D.D. the Chief Rabbi (the Rev. Dr. Adler), Viscount Duncannon, Lord Rowton, Lord Rothschild, Lord Iveagh, Lord Strathcona, Lord Farquhar, the Lord Mayor (Sir Marcus Samuel), the Chairman of the County Council (Sir John McDougall), the Chairman of the School Board for London (Lord Reay), the Governor of the Bank of England (Sir Augustus Prevost, Bart.), the President of the Royal College of Physicians (Sir W. S Church, Bart.), the President of the Royal College of Surgeons (Sir H. G. Howse), the Right Hon. Sir Savile Crossley, Bart., M.P., the Right Hon. Sir Joseph Dimsdale, Bart., M.P., Sir Trevor Lawrence, Bart., Sir John Aird, Bart., M.P., Sir Henry Burdett, K.C.B., Sir W. J. Collins, Mr. Sydney Buxton, M.P., Mr. J. G. Craggs, Mr. F. M. Fry, Mr. William Latham, K.C., Mr. J. Pierpont Morgan, Jr., Mr. Albert G. Sandeman, Mr. H. C. Smith, and Mr. Julius Wernher.

Letters of regret were reported from the following:—The Lord-Lieutenant of London (the Duke of Fife), the Duke of Norfolk, the Bishop of London, the Bishop of Rochester, Cardinal Vaughan, Lord Lister, the Postmaster-General (the Right Hon. J. Austen Chamberlain, M.P.), the Right Hon. C. Stuart Wortley, K.C., M.P., Mr. Thomas Burt, M.P., Mr. E. A. Hambro.

Lord Rothschild: Your Royal Highness, my Lords and Gentlemen,—It is now my duty to bring before you and before the Council the account of receipts and expenditure during the past year. Although as a rule a charity is never considered so flourishing as when it is in debt and has not the means of meeting its obligations, I think we may congratulate ourselves that King Edward's Hospital Fund is not in that position, and that during the last year it has met with large and generous support from all classes, and that we may look forward with confidence to the future. During the past year large gifts were given to His Majesty's Fund, and among those, I think I ought to specially mention the magnificent gifts of Lord Strathcona and Lord Mount Stephen amounting to £400,000 of securities, which will give the Fund, if I am not mistaken, at least £16,000 a year. Mr. Edgar Speyer has given securities to the value of £25,000, which will increase the annual income, I think, by £1,200 a year. Mrs. Lewis has placed at the disposal of the Fund £10,000 or £12,000 a year, which will go to swell the annual income, and enable us to look forward hopefully to the future.

The figures which I have the honour of presenting to your Royal Highness are considerably larger than we have ever had before. We received during the year £604,802. Of that amount £28,729 were annual subscriptions, £400,000 the value of the capital given by Lord Strathcona and Lord Mount Stephen, and £33,000 odd were donations at the discretion of the Council; £1,000 an annual grant from the trustees of London Parochial Charities for the maintenance of convalescent homes, £8,000 from the League of Mercy, £3,100 legacies, and £11,397 income from investments. The Coronation Gift amounted altogether to £118,663—that includes Mr. Speyer's gift, £25,000. The total receipts for the year were £604,802 18s. 5d. We distributed in gifts to the hospitals £101,000. The expenses were very small. The result of the whole year is as follows:—We left off with a balance of £76,724 10s. 2d. I suppose I shall be in order if I mention that of that £76,724, in all probability, £50,000 will be permanently invested in trustees' securities, leaving a balance of £26,724, which will be temporarily invested and earning interest. It must be a source of gratification to you, Sir, and to all concerned with the Council that the work of this great charity is carried on very efficiently and at very small cost to the subscribers. I have the honour to move that the accounts I have submitted be adopted.

The Lord Mayor: Your Royal Highness, my Lords and Gentlemen, I have very great pleasure in seconding the adoption of the accounts moved by Lord Rothschild. The good work that is being done by this Fund it is quite impossible to over-rate. The control over the management of the various hospitals, in seeing that they prepare their accounts in a proper form and that the money is not unduly wasted, is as great and as good a work as the distribution of the money itself. All who are interested in hospital work, and I suppose everyone is, must appreciate the fact that the time is coming when it will be absolutely necessary to stop the instituting of new foundations until those already existing have adequate support; and in this sense I find that the work being done by King Edward's Hospital Fund is a great and a good one. It gives the necessary *imprimatur* to those institutions who gain their support in appeals that they make to the public, and what is almost of equal consequence, it stops the stream of charity flowing to institutions which cannot prove to the satisfaction of the very able managers of this Fund that they are deserving of support from the public. I have very much pleasure in seconding the adoption of the accounts.

Sir John Aird: Your Royal Highness,—I have very great pleasure in supporting the resolution that has been proposed and seconded, and beg to offer my very best wishes for this noble work which was instituted some few years since by his Majesty the King, and which you will allow me to say has been so ably followed by yourself, Sir. There is no doubt that in this large city it behoves every good, right-thinking Englishman to do all in his power to uphold and help the benevolent institutions which do so much good in alleviating the sick and suffering poor. With very much pleasure I beg to support the resolution.

The Prince of Wales: My Lords and Gentlemen,—In supporting the adoption of the accounts and balance-sheet for the sixth year of the Fund, I am sure it is a matter of congratulation that during the year of the Coronation of the King, the founder of the Fund, it has achieved results far exceeding those hitherto obtained. In 1901 the contributions amounted to £53,000, and in 1902 they were, as Lord Rothschild has just told us, £604,000. The amount distributed in 1901 was £51,000, and in 1902 it was twice as much, or £101,000.

At our last meeting in November, I tendered our sincere thanks to the generous donors of large sums. In reviewing the whole results of the year, we now desire to express gratitude no less sincere to those who contributed smaller sums, to those who gave their shillings and pennies, and for the general sympathy extended to us by the public. Hitherto our account has shown a steady gradual progress. But this year's contributions show a more than tenfold increase. And while we recognise that these results were exceptional, and cannot be looked for again this year, still the hope which I ventured to express at the Council meeting last March, that our income should be increased by at least £50,000, was realised.

On December 31st last the investments held by the Fund may be reckoned as £662,000, producing, roughly speaking, £24,000 a year. To make up £50,000 a year from investments we want an addition of about £26,000 to our income, and to produce this at the present rate of Trust investments an addition of £870,000 to our capital would be necessary. But we may also count upon the reversion of £250,000 from the estate of the late Mr. Lewis, and I would remind you that through the generosity of Mrs. Lewis we are now receiving £10,000 a year. Am I too sanguine in hoping that before long the further amount of capital may be forthcoming? For the completion of this sum is a matter in which the King and I take the deepest personal interest.

I am sure you will agree with me that the accounts are most clearly drawn up. We have a special advantage in the fact that one of our honorary secretaries occupies an eminent position as a chartered accountant, and we fully recognise what an element of strength the Council derive from his presence in our body. (Hear, hear.)

I now put the resolution that the accounts and balance-sheet be adopted.

The motion of the adoption of the receipt and expenditure accounts and the balance-sheet was then put and carried unanimously.

Sir Savile Crossley read the report of the General Council for the year ending December 31st, 1902.

Lord Duncannon read the report of the Organising Committee.

The Prince of Wales: My Lords and Gentlemen,—In moving the adoption of the report, to which is appended the report of the organising committee, I wish to express my warmest thanks to all those whose combined efforts have brought about the gratifying results recorded in these reports.

I am desired by the King, the founder of the Fund, to congratulate the Council and all its workers upon its prosperous condition, and to assure them that his Majesty watches the growth and vitality of the Fund with as much interest and satisfaction as he did when President. The greatest credit is due to the thorough painstaking work of the visiting committee. It has been largely augmented, and more members will still have to be added as the work increases. On this systematic visiting by both lay and medical representatives is based that principle of investigation which is slowly but surely securing the public confidence in the Fund.

I am well aware that the people of this country are ready to support the hospitals provided that they receive clear proof that the money given by them is well spent. Anyone who looks into the management of the Fund will see that no trouble is spared to lay out the money entrusted to it to the best possible advantage. I am glad to see the other hospital funds base their policy on similar lines, and are working side by side with us with simultaneous success.

The King, in his original letter in 1897, stated that our endeavours should be to raise an annual sum of from £100 to £150,000. But every year progress is being made in medicine and surgery, which, with an ever-increasing population, means additional expenses incurred by our hospitals. Therefore, it is my earnest hope that our efforts will be redoubled to make generally known the work and object of the Fund, in view of the fact that as time goes on increasing demands may be made for our assistance.

This year it is settled to extend our area so as to take in the limits of the London County Council where that limit exceeds the seven-mile radius from Charing Cross. This will include many poor districts, especially those in the south-east of London. In considering the ever-growing demands of the Metropolitan Hospitals, we must not overlook the fact that 23 per cent. of their patients come from the country. I earnestly trust that the liberal contributions to the Coronation gift which were received last year from many of the cities and towns throughout the country, may be regarded not only as a recognition of this fact, but also as an assurance of regular future support from the provinces.

This year's report alludes to that highly important question of the amalgamation and removal of certain hospitals I am extremely glad to learn that the committee's efforts have so far received considerable sympathy and support. For I am convinced that the general public will appreciate the high sense of duty which has actuated representatives of these institutions to subordinate their private interests in order to consider those of the entire hospital system at large.

Hitherto we have confined our attention to the hospitals, while the convalescent homes have only been assisted from the grant of £1,000 a year entrusted to us by the London Parochial Charities. But I look forward to the day when both the hospitals and the convalescent homes attached to them will participate more fully in the benefits of the Fund. The distinguished professional authorities present here will bear me out when I say that these homes are absolutely essential to ensure complete recovery of many cases treated in our hospitals.

The work of the League of Mercy has steadily increased, thanks to the unflagging energy of its honorary officials, to whom we offer our cordial thanks for the substantial addition which they have been able this year to place to the credit of the Fund.

Allusion has already been made to the Coronation Gift, and I am sure the Council heartily reciprocate the gracious words of congratulation which fell from the King on the occasion of his receiving the Gift at the hands of the Organising Committee.

In conclusion, I beg to express my deep appreciation of the valuable services rendered during the past year by the General Council, the honorary treasurer, the honorary secretaries, the visiting committee, the distribution committees, and other honorary officials. I am well aware of the heavy sacrifices of time and of other work which have been involved by their labours, and I offer them my most grateful thanks, and trust that they will again be kind enough to serve for another year in their respective capacities. (Hear, hear).

I now beg to move the adoption of the Report.

The Right Hon. Sir Joseph Dimsdale, Bart., M.P.: May it please your Royal Highness, it affords me the greatest pleasure to second the adoption of this report, and I hope it will not be considered impertinence on my part if I congratulate you on behalf of myself and those around this table on the great success of your first year as president. I hope we are only at the commencement of the development of this great and noble work.

Lord Reay: I have great pleasure in supporting this resolution. The report shows that there is a general feeling among the public that the growing need which this work shows to exist is met by the committee, over which your Royal Highness presides, with so much vigour and in a way that commends itself to the public. We have every guarantee that the work of the Fund is widely progressive, and that your Royal Highness may be well satisfied with what has already been accomplished.

The report, on the motion of the Prince of Wales, was received and adopted.

Lord Strathcona: Your Royal Highness, my Lords and

Gentlemen,—I am sure you desire, and we also know it is the desire of everyone who takes an interest in the magnificent work done by the Metropolitan hospitals, to give an expression of our thanks to his Royal Highness for the great interest he takes in this Fund—the work begun on the initiation of His Majesty, and happily taken up by his Royal Highness. And I may be permitted perhaps to say this one word. I think that what has been already said with regard to this work is apposite: it is not the actual grant given to each hospital that is valuable, so much as the fact that the grant is only given after a thorough examination. We know that as a whole the hospitals of the Metropolis have done for many years, and continue to do, very great and good work, but it has not been found that they have been all equally perfect at all times. When it is known that it is only to those who endeavour to conduct and administer the affairs of their hospitals in the best way that any assistance will be given from the Fund, it cannot be otherwise than a great advantage, and one which will make them still more perfect. The work being done under the auspices of His Royal Highness and by those whom he nominates as treasurers and otherwise, must induce many, not only within the metropolis but throughout the country, to come forward even more than in the past to assist this great work, knowing and having the assurance that it will be administered in the best possible way.

I know it is your wish that we should express to His Royal Highness our sense of warm gratitude for all he has done and is doing, and I therefore have the honour to move a vote of thanks.

Sir Henry Burdett: Your Royal Highness, my Lords and Gentlemen,—I have very great pleasure in seconding the vote of thanks to our President, the Prince of Wales, as I feel it to be a special privilege to have the honour of seconding this vote, because we have at the head of this Fund one who has set us all an example of personal service, and has had a material influence on the work we have individually undertaken in the cause of the hospitals. I should like to say it affords me great pleasure to be able to bear testimony to the fact that this Fund has in the organisation of the League of Mercy the means of obtaining the help of the humblest citizens of London. Your assistance as our President has been a great help to all of us who have tried to do our best to help the hospitals through the King's Fund. I have great pleasure in seconding a most cordial and hearty vote of thanks to your Royal Highness as president of the King's Hospital Fund.

Sir John M'Dougall: Your Royal Highness, my Lords, and Gentlemen, may I, as representing London in the shape of the London County Council, have the honour to support this resolution of a vote of thanks to your Royal Highness? The matter of the hospitals is everything to the poor. They flock to the hospitals because they know they will be properly and kindly treated. The hospitals have been well managed, and are even coming more to the front because of the supervision of this Committee, which is looking into the details and management. I know sometimes we think we do pretty well without control, but the suggestions that come from this Board are always considered by the Hospital's Board of Management and always with advantage. Therefore I have the greatest pleasure in supporting this vote of thanks to your Royal Highness for presiding over this Board which will continue to alleviate distress and suffering and pain.

The Prince of Wales: My Lords and Gentlemen, I very much appreciate the kind words which have been said by Lord Strathcona, Sir Henry Burdett, and Sir John M'Dougall in proposing this vote of thanks. I am sure I do not wish to be thanked for anything I may have done, and I am afraid I do not deserve it. But I can only say that whatever I do is a most pleasant duty, and certainly anything connected with the hospitals of London and the alleviation of the poor and suffering is very near my heart. I am very pleased to meet you all here to-day, gentlemen, and I know I can count on your most valuable eloquence and support.

The proceedings then terminated.

